# Sustainable Prevention of Cholangiocarcinoma Through Community Participation in a High-incidence Area in Thailand

**DOI:** 10.31557/APJCP.2020.21.3.777

**Published:** 2020-03

**Authors:** Nopparat Songserm, Somkiattiyos Woradet, Banchop Sripa, Akhtar Ali

**Affiliations:** 1 *Department of Community Health, Faculty of Public Health, Ubon Ratchathani Rajabhat University, Ubon Ratchathani, *; 2 *Department of Public Health, Faculty of Health and Sports Science, Thaksin University, Phattalung, *; 3 *Department of Pathology, Faculty of Medicine, Khon Kaen University, Khon Kaen, *; 4 *WHO Collaborating Centre for Research and Control of Opisthorchiasis (Southeast Asian Liver Fluke Disease)/Tropical Disease Research Center (TDRC), Thailand, *; 5 *Department of Biological Science, The University of Tulsa, Oklahoma, United State of America. Editorial Process: Submission:12/07/2019 Acceptance:03/04/2020*

**Keywords:** Opisthorchiasis, cholangiocarcinoma, prevention, community participation

## Abstract

Opisthorchiasis and cholangiocarcinoma (CCA) are major public health problems in Northeast Thailand, especially in Ubon Ratchathani, which are the alluvial plains. Those with poor food habits are mostly at risk of having diseases. This study was a participatory action research (PAR) aimed to define the models/plans for CCA prevention. The samples consisted of 40 community representatives. The data were collected by the PAR method. Qualitative data were analyzed by content analysis. The samples coordinately analyzed and prioritized the problems and presented information to the community. The plans consisted of (1) cultivating the right values and behaviors of eating food safe from CCA in children, (2) supervising the shops not to sell foods that are the main risk factors for CCA, (3) supporting the community leaders to act as good role models to the people, (4) assigning every housewife to cook clean and safe food from CCA, and (5) encouraging the villagers to have knowledge and awareness, which can protect them from CCA. After the community designed these plans, responsible persons were assigned to implement them. Two years later, researchers evaluated the outcome. The average scores on knowledge and attitude toward CCA were significantly higher than before implementation. For the impact of projects, prevalence of opisthorchiasis in 2016 was significantly lower than that in 2014. CCA prevention by community participation employed a combination of quantitative and qualitative studies. This study has been successful and sustainable since the community has human resources, budget, and appropriate management of the project.

## Introduction

Cholangiocarcinoma (CCA) is still a major public health problem among Thais, especially in the northeast, which is the area with the highest incidence of disease in the world (Vatanasapt et al., 1990). Based on data from the National Cancer Institute in 2001-2003, the incidence of CCA in Thailand was 38.6 and 14.6 per 100,000 in males and females, respectively (Khuhaprema et al., 2010). In 2004-2006, the trend was likely to increase in both males and females to 42.8 and 18.2, respectively (Khuhaprema et al., 2012). When separately described by region, the northeast had the highest incidence rates, which were 83.4 and 39.6 per 100,000 in males and females, respectively. Ubon Ratchathani had the highest incidence of CCA compared to other types of cancer. In 2001-2003, the incidence rates were 74.9 and 34.7and increased in 2004-2006 to 80.6 and 41.6 per 100,000 males and females, respectively (Khuhaprema et al., 2010; Khuhaprema et al., 2012). According to the database of Ubon Ratchathani Cancer Center, Region 7, the first three areas with the highest death rate from liver and bile duct cancers were the Khueang Nai District, Phibun Mangsahan District, and Muang District, with rates of 11.84, 6.77 and 4.35 per 100,000 persons, respectively. In the Muang District, the Nong Bo Sub-district surrounded by the Se Bai River, Chi River, and Mun River was the area with the highest death rate from opisthorchiasis and CCA (Ubon Ratchathani Cancer Center, 2010).

The factors contributing to the high incidence of CCA are consumption behaviors such as (1) eating raw or uncooked fish, such as Koi Pla (raw fish salad) or Pla Jom (pickled fish), which is a source of liver fluke infection; (2) eating fermented food, such as fermented fish and soured fish, which are sources of liver fluke infection and nitrosamines that are significant causes of CCA (Srivatanakul et al., 1991; Haswell-Elkins et al., 1994; Songserm et al., 2012); and (3) drinking alcohol, which is another important risk factor for CCA (Shin et al., 1996; Uttaravichien et al., 1999; Chalasani et al., 2000; Chernrungroj, 2000; Honjo et al., 2005). Therefore, to reduce the incidence of CCA in high-incidence areas, it is necessary to reduce the risk factors for CCA, as well as to correct the behaviors of disease prevention and control by encouraging people to have knowledge, health beliefs, and correct behaviors to avoid various risk factors. These are the most sustainable prevention and control methods for CCA.

The community needs to be involved in the problem-solving process to encourage people to have sustainable knowledge, health beliefs, and the right behaviors to avoid various risk factors for CCA. That is, the community is required to participate in the study and analyze the problems, as well as plan, implement, obtain benefits, follow up, and evaluate the project together. Therefore, participatory action research (PAR) is the most appropriate form of research, since the community pursues the ideas, resources available in the community, and solutions to problems simultaneously for themselves. This will result in a long-lasting project because everyone will be involved. The community cooperates in thinking and operating and has a feeling of ownership of the project together. Therefore, this research aimed to investigate the models and plans that people in risky areas have for the prevention and control of CCA through community participation.

## Materials and Methods

*Study design*

This PAR study was used by people in the community to determine the models and plans using personnel and resources within the community combined with those of government agencies and organizations inside and outside the research area to obtain the models and plans that people can cooperatively solve for preventing and controlling CCA. The details of the study design have been published previously (Songserm et al., 2015; Songserm et al., 2016). This research was approved by the Ubon Ratchathani Rajabhat University Ethics Committee for Human Research, based on the Declaration of Helsinki and the ICH Good Clinical Practice Guidelines.

*Determination of the study area*

**Figure 1:**
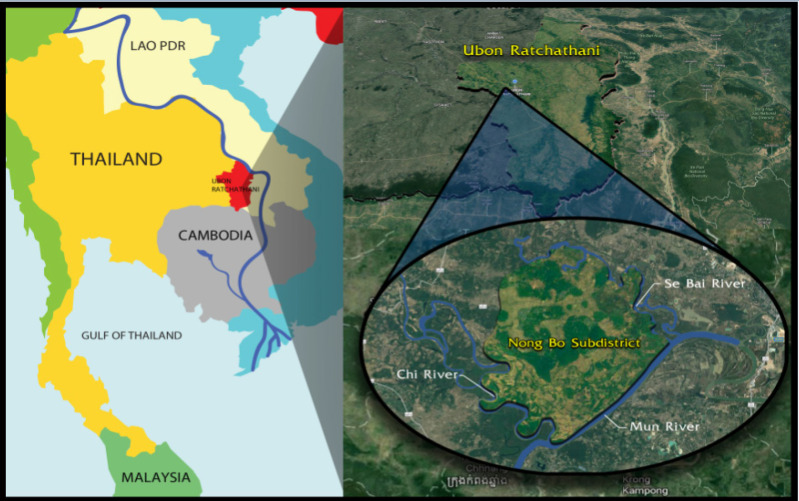
Determination of the Study Area (Source: Google Maps)

The Nong Bo Sub-district, Muang District, Ubon Ratchathani was selected as the study area since the prevalence of liver fluke infection was high, and the incidence of CCA was the highest (Ubon Ratchathani Cancer Center, 2010). This might be because of many contributing factors. For example, fresh water sources are abundant, such as the Se Bai River, Chi River, and Mun River, and there are many streams and swamps Figure 1. This factor encourages most people in the area to be fishermen, and there is a high chance of catching freshwater fish for consumption of raw or uncooked freshwater fish.

*Population and samples*

The population and samples in this study consisted of representatives of the villagers, community leaders, monks, youth leaders, housewife leaders, elderly club leaders, and developers, including public health officers, teachers, Tambon agriculture officers, the president of the Tambon Administrative Organization, and the members of the Tambon Administrative Organization. Therefore, there were 40 samples in total. The samples were selected by the researchers, community leaders, and networks. Written informed consent was obtained from all participants.

*Research process*

There were 3 steps as follows.

1. Step 1 (Preparation): This step was important for building good relationships, understanding and trust among the researchers, community leaders, people and government officials in the area. It was also the stage of collecting important information about the area such as maps, social conditions, geography, fresh water resources, occupation, and daily life so that researchers can understand and absorb the information for the community and truly understand the way people live in the community. It also made people in the area trust the researchers and all the relevant working groups.

2. Step 2 (Operation): This step was the main activity of the research. The purpose was to provide the villagers, community leaders, and developers who were government officials in the area a plan to solve the problem of CCA of the community. The researchers were just the organizers of the meeting process. The research team invited the 40 local stakeholders to attend the meeting to recognize the problems, analyze or criticize the causes of the problems. and to plan for problem solving, as well as to create the project, fund a budget and determine those who were responsible for community activities. The activities were as follows. 

1) Studying and analyzing the problems within the community. In this process, the team of researchers and research assistants collected data using a questionnaire from 906 households, covering 13 villages in the research area. This provided the quantitative data presented in the previous study (Songserm et al., 2015; Songserm et al., 2016) together with information from the process of entering the community and building relationships with the community. All the data were analyzed and presented in a simple way. Local language, videos, maps, slides, and boards were used to present the information to help the participants perceive issues related to the common risk factors for CCA. 

2) Prioritizing the community’s problems. When all the participants had deeply learned and understood the problems together, the conference leaders led all the participants in prioritizing the problems. They considered the size and severity of the problems, the recognition of the problems by the villagers, and the difficulty in solving the problems. Each participant had the right to vote independently. The scores were counted, and the problems were prioritized from the most to the least problematic. Everyone analyzed and discussed the results together again before making a summary and selected the five most important problems for the next planning process.

3) Corporately planning the plan. When the problems were prioritized, and the top five important problems were selected, the conference leaders divided the participants into five subgroups according to the need to solve the problems of each person to allow those who are directly affected by the problems to plan to solve the problems together. They also had to plan, define activities and schedules, specify the time for organizing activities, determine the budget source, identify the responsible persons or agencies by focusing on self-reliance in the area and using human resources. The speakers and the researchers were the consultants or mentors in writing the plan. The members in the subgroups freely discussed the issues and created a plan. Once they finished planning the work, each subgroup assigned the representatives to present the plan at the meeting to allow all participants to know and discuss the plan or make suggestions. 

4) Coordinating and implementing the plans: Once the plans for solving the problem of CCA had been obtained by community participation, they were implemented. The researchers provided the areas that had been planned for the implementation. The researchers observed and encouraged the villagers to organize activities and supported their academic assistance and other resources to strengthen the community and provide self-sustainability.

3. Step 3 (The follow up and evaluation process). Step 3 consisted of (1) the evaluation of the outcome, including the knowledge of and attitude toward opisthorchiasis and CCA, and (2) the evaluation of the impact on the prevalence of liver fluke infection. 

*Data analysis*

1. Quantitative data analysis: This consisted of an analysis of the number of plans that were implemented to prevent CCA, the level of knowledge and attitude about opisthorchiasis and CCA, and the prevalence of liver fluke infection before and after conducting the research. Descriptive statistics, including frequency, percentage, mean, standard deviation, and prevalence were used. 

2. Qualitative data analysis: The analysis of PAR was conducted to understand and interpret the content of the information using content analysis. This was simultaneously done with the data collection of each stage of the research. It was conducted by researchers and research assistants who lived in the area and who were knowledgeable and understanding of the context of the study.

## Results

The survey consisted of three parts: (1) general information of people and context of the area; (2) risk factors for CCA; and (3) health determinants related to CCA. The results in the first phase of the study were used to plan the models for CCA prevention and control. The research team proceeded to prepare the people in the community and establish a cooperative network. The research team built a relationship and learned about the community. At the same time, the research team tried to persuade people in the community to attend a participatory workshop to find the solution to the problem of CCA. After this step, the research team started the operation. This was the main activity of this research. The purpose was to provide the villagers, community leaders, and developers who were government officials in the area a plan to solve the problem of CCA in the community. The researchers were just the organizers of the meeting. The results obtained from the sub-activities of PAR were as follows.

1. Studying and analyzing the problems within the community. In this process, this was a community study for sharing and learning in social dimensions in the community, geography, culture, and way of life, and the information related to risk factors for CCA. At the meeting, it was stated that the community still had patients with opisthorchiasis and CCA. The causes of the diseases can be concluded as follows. (1) The villagers liked to drink alcohol, especially beer and white whisky. (2) There was liver fluke infection. (3) The villagers refused to check for liver flukes. (4) The villagers often used praziquantel unnecessarily and did not consult doctors. (5) The villagers ate Koi Pla (raw fish salad) or Pla Jom (pickled fish). (6) The villagers ate soured fish. (7) Most villagers ate uncooked fermented fish. The villagers argued that “cooked fermented fish is not delicious.” According to the results from the meeting, the researchers took the problems and prioritized them in the next step.

2. Prioritizing the community problems. When all participants deeply learned and understood the problems together, the speakers and conference leaders led all the participants to prioritize the problems. The size and the severity of the problems, the acceptance and recognition of the problems by the villagers, and the difficulty in solving the problems were considered. Each participant had the right to vote independently. The scores were counted, and the problems were prioritized from the highest priority to the least priority. Everyone analyzed and discussed the list together again before making a summary and selected the five most important problems. The problems can be summarized as follows: 1) eating uncooked fermented fish, 2) eating Koi Pla (raw fish salad) or Pla Jom (pickled fish), 3) alcohol consumption, 4) repeatedly using praziquantel, and 5) not checking faces for parasites. These were behavioral health problems causing opisthorchiasis and CCA and remained major problems for people in the area. The research team then brought these five issues to the planning committee to address the problems using PAR in the next step. 

3. Analysis of potential problems in the PAR process and solutions. This step was intended to allow the villagers, the leaders, and the developers who were government officials in the area to plan together based on PAR for solving the problem of CCA in the community. The researchers were just the organizers of the meeting. The research team invited 40 local stakeholders to attend the meeting to recognize the problems, analyze or criticize the causes of the problems, and to plan for problem solving as well as to create the project and a budget and determine those who were responsible for community activities. 

4. Planning: After prioritizing the first five problems with the community participation, the research team, who conducted the meeting, allowed all the participants to set plans for the community using personnel and resources within the community and those of government agencies and organizations inside and outside the research area to get models or plans that people can cooperatively use to solve the problems of CCA control and prevention with community participation. Finally, they planned to monitor and evaluate the project and activities. The outputs included five plans Table 1.

After the community obtained the plans to prevent and control CCA through community participation, the responsible persons were assigned to implement the plans. The researchers did not play any roles after this stage. It was the role of the community to participate in the implementation, obtain benefits, follow up, and evaluate the plans. Two years later, the researchers came back to evaluate the operation as follows. 

1) The evaluation of the outcome. In this study, the scores of the knowledge of and attitude toward opisthorchiasis and CCA were used as indicators of evaluation. It was found that in terms of knowledge, after the implementation of the project, the scores of the samples were statistically significantly higher than those before the implementation of the project with a mean score of 1.24 (p <0.001). Similarly, the scores on the attitude toward opisthorchiasis and CCA of the community after the implementation of the project were significantly higher than those before the implementation of the project with the mean score of 1.81 (p<0.001) Table 2.

2) The evaluation of the impact. In this study, the prevalence of opisthorchiasis was measured after 2 years of implementing the plans. In 2016, the prevalence of opisthorchiasis in the samples significantly decreased from the 2014 by 11.95 percent (p <0.001) Table 3.

**Table 1:** The Details of the Plans for Cholangiocarcinoma Prevention of People in a High-incidence Area of Ubon Ratchathani, Thailand

Plans	Objectives	Target groups	Evaluation	Responsible people
1. Adding the content of opisthorchiasis and CCA in elementary and secondary schools	To cultivate the right values and behaviors for children and encourage them not to eat raw fish	Kindergarten / elementary / secondary students	- Teaching plans - Asking from s tudents	- Six elementary schools - One secondary school in the area
2. Launching community rules by directing shops or restaurants to cook all kinds of food before selling it	To prevent people from eating Koi Pla (raw fish salad), uncooked fermented fish and soured fish	Every shop or restaurant in the community	Random survey	Tambon Administrative Organization
3. Asking for cooperation from the community leaders and volunteers to serve as role Models of not eating raw fish or raw foods and not drinking alcohol	To be good role models before teaching others	All community leaders and Health volunteers in the community	- Observing or tracking behaviors - Asking the Villagers	- All community leaders - All health Volunteers in the community
4. Training or encouraging housewives to cook clean food	To make every housewife cook cooked and safe food for everyone	Housewives or cooks of all households	- Survey using questionnaire - Observation	- Two Health Promoting Hospitals - Health volunteers
5. Providing knowledge about the risk of opisthorchiasis and CCA to all villagers in the community, such as (1) arranging an audio program every Wednesday evening and Friday or (2) organizing a contest of family away from opisthorchiasis and CCA	To encourage the villagers to have knowledge and awareness	All villagers	- Observing or tracking behaviors - Asking the villagers - Having prizes for families practicing to prevent such diseases	- Tambon Administrative Organization - Two Health Promoting Hospitals

**Table 2:** The Comparison of Knowledge of and Attitude Toward Opisthorchiasis and Cholangiocarcinoma before and after Participation in the Research Project in a High-incidence area of Ubon Ratchathani, Thailand

Factors	No.	Mean	S.D.	Mean difference	95% CI	P-value*
Knowledge						
Pre-test	426	6.80	1.77	1.24	1.03-1.44	<0.001
Post-test	556	8.04	1.52			
Attitude						
Pre-test	426	24.57	3.24	1.81	1.45-2.18	<0.001
Post-test	556	26.38	2.60			

*P-value for t-test

**Table 3:** The Comparison of the Prevalence of Opisthorchiasis before and after Participating in the Research Project in a High-incidence Area of Ubon Ratchathani, Thailand

Year of survey	No. of samples	No. of infections	Prevalence (95% CI)	Prevalence difference (95% CI)	P-value*
2014	224	39	17.41%	11.95%	<0.001
			(12.44 – 22.38)	(6.46 – 17.43)	
2016	366	20	5.46%		
			(3.14 – 7.79)		

*P-value for Z-test

## Discussion

The purpose of this research was to explore the models or plans that people in this risky area can participate in for the planning, prevention, and control of CCA through community participation. PAR was employed in a high-incidence area. The research team introduced the quantitative data obtained from the first phase of the study. It can be concluded that most of the samples in first phase of the study were females (63.69%). The average age was 51.39 ± 10.29 years old. Agriculture was the main occupation at 85.98%. The educational level was primary school (75.17%). The average family income was 15,000 Baht, and 62.14% of families achieved that level of income. Persons with a marital status of married or living together made up 78.92% of the population. The study of prevalence and the factors related to the risk of CCA in the area were smoking, alcohol drinking, history of having opisthorchiasis, the use of praziquantel, consumption of raw fish, and foods containing nitrosamines (Songserm et al., 2015; Songserm et al., 2016). The data were then used as a starting point to identify the models or plans for the prevention and control of CCA through community participation in the second phase. 

The models or plans for the prevention and control of CCA through community participation started by studying and analyzing the problems in the community, prioritizing the problems, analyzing the problems and determining the solutions, creating plans, and presenting the data at the community meeting. These processes were conducted through PAR with the community representatives. From this meeting, the community representatives identified and prioritized the problems that were the causes of opisthorchiasis and CCA in the community. These were consistent with the problems that the researchers had surveyed in preliminary data from the villagers in 13 villages earlier (Songserm et al., 2015; Songserm et al., 2016). It also led to the planning process to address the problems more easily, as both the leaders and the community had the same opinion. From planning of the subgroups, five work plans were obtained, and they were distributed to different target groups as well as the people responsible. The five works plans are as follows: (1) cultivating the right values and behaviors of eating foods that are free from opisthorchiasis and CCA for children and youth by asking for the cooperation of primary and secondary schools in the community to add or include instructional content about the diseases in teaching, (2) instructing stores not to sell foods that are major risk factors for opisthorchiasis and CCA by launching community rules, (3) supporting the community leaders and healthcare volunteers in acting as good role models for the people in the community by consuming foods that are safe from opisthorchiasis and CCA, (4) assigning housewives of all the households to cook clean food safe from opisthorchiasis and CCA, and (5) encouraging the villagers to develop knowledge and awareness so that they can carry on their practice of avoiding opisthorchiasis and CCA. The results of this study were consistent with those of the study to explore the ways to prevent and control opisthorchiasis and CCA in Khon Kaen Province of Thailand in terms of using action research to prevent opisthorchiasis (Wongba et al., 2011), community process development (Duangsong et al., 2013), and host-based opisthorchiasis control (including people, dogs and cats) and the environment at Lawa Lake in Khon Kaen, Thailand (Sripa et al., 2015). This was also consistent with studies on chronic diseases and other types of cancer conducted by Promthet et al. (2012) and Saranrittichai et al. (2012), which evaluated the health and knowledge of people attending public health training programs in the northeast of Thailand. 

Two years later, the research team came back to evaluate the operation using knowledge and attitude scores on opisthorchiasis and CCA as indicators of the operation. It was found that after the implementation, the scores of the samples were significantly higher than those of before the implementation of the project. This indicated that PAR was effective in allowing the people in a risky area to have more knowledge of and a better attitude toward the prevention of opisthorchiasis and CCA than those before doing research. This is particularly useful in the public heath dimension where there are few employees or staff. We can transfer knowledge to many people effectively. This is an important and necessary issue in rural areas where people lack human resources, budgets, and implementation tools. This was consistent with the study on the evaluation of health education provided for health officers in Khon Kaen, Thailand (Promthet et al., 2012).

For the evaluation of the impact, the prevalence of opisthorchiasis was used as an indicator. It was found that after two years of operation of the plans, the prevalence of opisthorchiasis in the samples decreased with statistical significance. This noted that PAR was effective in reducing or stopping people in a risky area from eating raw fish. As a result, it obviously decreased liver fluke infection. This was in accordance with the research conducted by Sripa et al. (2017) who conducted a study in an area with a high incidence of opisthorchiasis and CCA: Lawa Lake, Khon Kaen, Thailand. The EcoHealth approach was employed and led to the development of a sustainable opisthorchiasis control program. 

In conclusion, the prevention and control of opisthorchiasis and CCA by community participation using a combination of both quantitative and qualitative studies started by studying the prevalence of risk factors, knowledge, health beliefs, and behaviors in CCA prevention and community participation from the public. Then, the factors related to the overall risk level for CCA were studied. When the knowledge was obtained, it was presented to community representatives at a meeting to determine the models and plans for the prevention and control of CCA using PAR. This study has been successful and sustainable since the community has human resources, a budget, and appropriate management for the project.
